# Isolation and Identification of the Microbiota of Danish Farmhouse and Industrially Produced Surface-Ripened Cheeses

**DOI:** 10.1007/s00248-012-0138-3

**Published:** 2012-12-07

**Authors:** Klaus Gori, Mia Ryssel, Nils Arneborg, Lene Jespersen

**Affiliations:** Department of Food Science, Food Microbiology, Faculty of Life Sciences, University of Copenhagen, Rolighedsvej 30, 1958 Frederiksberg C, Denmark

## Abstract

For studying the microbiota of four Danish surface-ripened cheeses produced at three farmhouses and one industrial dairy, both a culture-dependent and culture-independent approach were used. After dereplication of the initial set of 433 isolates by (GTG)5-PCR fingerprinting, 217 bacterial and 25 yeast isolates were identified by sequencing of the 16S rRNA gene or the D1/D2 domain of the 26S rRNA gene, respectively. At the end of ripening, the cheese core microbiota of the farmhouse cheeses consisted of the mesophilic lactic acid bacteria (LAB) starter cultures *Lactococcus lactis* subsp. *lactis* and *Leuconostoc mesenteorides* as well as non-starter LAB including different *Lactobacillus* spp. The cheese from the industrial dairy was almost exclusively dominated by *Lb. paracasei*. The surface bacterial microbiota of all four cheeses were dominated by *Corynebacterium* spp. and/or *Brachybacterium* spp. *Brevibacterium* spp. was found to be subdominant compared to other bacteria on the farmhouse cheeses, and no *Brevibacterium* spp. was found on the cheese from the industrial dairy, even though *B. linens* was used as surface-ripening culture. Moreover, Gram-negative bacteria identified as *Alcalignes faecalis* and *Proteus vulgaris* were found on one of the farmhouse cheeses. The surface yeast microbiota consisted primarily of one dominating species for each cheese. For the farmhouse cheeses, the dominant yeast species were *Yarrowia lipolytica*, *Geotrichum* spp. and *Debaryomyces hansenii*, respectively, and for the cheese from the industrial dairy, *D*. *hansenii* was the dominant yeast species. Additionally, denaturing gradient gel electrophoresis (DGGE) analysis revealed that *Streptococcus thermophilus* was present in the farmhouse raw milk cheese analysed in this study. Furthermore, DGGE bands corresponding to *Vagococcus carniphilus*, *Psychrobacter* spp. and *Lb. curvatus* on the cheese surfaces indicated that these bacterial species may play a role in cheese ripening.

## Introduction

Cheeses harbour a complex microbiota characterised by a succession of different microorganisms during milk coagulation and ripening [24]. During cheese ripening, lactic acid bacteria (LAB) starter cultures (e.g., mesophilic *Lactocococcus lactis* or thermophilic *Streptococcus thermophilus*) metabolise residual lactose and citrate to different aroma compounds [17]. Later, LAB starter numbers decrease with cell death and their subsequent lysis results in release of intracellular peptidases involved in proteolysis of peptides to free amino acids [8, 31, 54]. Several free amino acids are flavour compounds themselves, but more important free amino acids are precursors of other cheese flavour compounds including ammonia, carbonyl and sulphur compounds. Non-starter LAB (homo- and heterofermentative lactobacilli and pediococci) either present as indigenous milk microorganisms, contaminants or added as ripening cultures will grow during ripening and in most cases enhance flavour intensity [12].

Surface-ripened cheeses are characterised by an additional ripening from the cheese surface to the interior due to the activity of both yeasts and bacteria on the cheese surface [7]. During the initial ripening period, yeasts (primarily *Debaryomyces hansenii* for semi-soft cheeses and additionally *Geotrichum candidum* for soft cheeses) and coagulase-negative staphylococci (*Staphylococcus equorum*) are present [3, 21, 26]. Generally, *D*. *hansenii* and staphylococci on cheese surface are assumed to originate from the cheese brine, which often is not changed or pasteurised between salting of different batches [2, 3, 42]. For *D*. *hansenii*, Petersen et al. [46] showed that the dominating *D*. *hansenii* strain on cheeses of the Danish Danbo type did not originate from the added ripening culture, but from the dairy housemicrobiota present in the ripening room. *D*. *hansenii* is important during cheese ripening as it assimilates lactate and produces alkaline metabolites such as ammonia thereby increasing pH of the cheese surface [23, 46], which enables the growth of the less acid tolerant bacterial microbiota, primarily Gram-positive coryneforms (*Brevibacterium* spp., *Corynebacterium* spp. and *Microbacterium* spp.) [4]. In addition, subpopulations of bacteria such as Gram-positive *Marinilactibacillus* spp. and Gram-negative *Halomonas* spp., *Vibrio* spp. and *Proteus* spp., and bacteria of the *Enterobacteriaceae* family have been reported to occur on cheese surfaces [14, 15, 25, 34, 35, 41, 50]. The presence of Gram-negative bacteria was first hypothesised to be indicative of hygienic problems. However, more recent results have shown that they produce important cheese flavour compounds and thus might contribute positively to the cheese ripening process [11]. Several studies have characterised the cheese microbiota by both culture-dependent methods and -independent methods [43]. Following culture isolation, identification has been based on either macro- and micromorphological examinations and by genotypic identification based on DNA restriction, amplification and sequencing. Furthermore, many culture independent methods, e.g., denaturing gradient gel electrophoresis (DGGE) have been used to study microbial diversity in cheeses [27]. Most recently one study has included the pyrosequencing technique for identification of cheese microbiota [38].

During the last decade, increasing interest in high-quality cheeses produced at Danish farmhouses has resulted in an increasing number of Danish farmhouses producing a large variety of cheeses, of which many are surface-ripened. Contrary to the microbiota of, in particular, German and French surface-ripened cheese varieties, the microbiota of Danish surface-ripened cheeses have only been investigated to a limited extent [37, 38, 46].

The aim of the present study was to investigate the microbiota of three surface-ripened cheeses produced at three individual Danish farmhouses and one surface-ripened cheese produced at one Danish industrial dairy. Investigations of the cheese microbiota composition are highly relevant, as the cheese microbiota together with rennet and indigenous milk enzymes plays an important role for both the flavour (aroma and taste) and textural properties of the final product. To our knowledge, this is the first study, which simultaneously identified the cheese microbiota including both bacteria and yeasts in samples taken separately from the cheese interior and surface by both culture-dependent and -independent approaches.

## Materials and Methods

### Cheese Manufacture

The present study was conducted on three surface-ripened cheeses produced at three individual Danish farmhouses (dairies A, B and C) and one surface-ripened cheese produced at one Danish industrial dairy (dairy D). Cheeses from dairy A and C were of the Havarti type, whereas cheeses from dairy B and D were of the Danbo type). All cheeses were made with mesophilic LAB starters, and except for the cheese from dairy A made of pasteurized milk. After brining, cheeses from dairies A, C and D were smeared with *Brevibacterium linens*, whereas no commercial ripening cultures were used for the cheese from Dairy B.

### Sampling and Isolation of Microbiota

For the cheeses from dairies A and C, sampling took place at the end of ripening, which for both cheeses was 12 weeks, whereas for the cheeses from dairy B and D, sampling took place prior to the washing and paraffin treatment, which was after 6 weeks of ripening. One sample from each cheese was analysed. Ten grams of cheese from the surface (depth ~ 4 mm) and the interior, respectively, was removed using a sterile scalpel, and 2 % (w/v) trisodium citrate was added to yield a 1:10 dilution in stomacher bag. The mixture was homogenized using a Stomacher for 2 min at medium speed. From this dilution, 10-fold dilutions were prepared in 0.9 % (w/v) NaCl. The interior bacterial microbiota was enumerated on M17 with 1.0 % glucose (GM17) incubated for 3–4 days aerobically at 30 °C and 37 °C, respectively, and on MRS (pH 6.2 and 5.4) incubated for 3–4 days anaerobically at 30 °C and 37 °C, respectively. The surface bacterial microbiota was enumerated on tryptic soya agar (TSA) with 0.0 % and 4.0 % (w/v) NaCl, respectively, incubated for 10–12 days aerobically at 30 °C. All media for bacterial enumeration were added 0.2 % (w/v) sorbic acid (Merck) and 0.1 % (w/v) cycloheximide (Merck) to suppress growth of moulds and yeasts. The interior and surface yeast microbiota was enumerated on Malt Yeast Glucose Peptone (MYGP) agar composed of 3.0 g of malt extract (Difco), 3.0 g yeast extract (Difco), 10 g of glucose (Merck), 5.0 g Bactopeptone (Difco) and 15 g of agar (Difco) per litre of distilled water, pH 5.6, incubated for 5–8 days aerobically at 25 °C. MYGP was added 100 mg/l chloramphenicol and 50 mg/ml chlortetracycline (Sigma, St. Louis, MO, USA) to suppress bacterial growth. Twenty to forty bacterial and yeast colonies were selected from countable plates and were purified by re-streaking twice on the appropriate media. For long-term storage, purified isolates were stored at −80°C in appropriate media containing 20 % (w/v) glycerol.

### Chemical Analyses

Moisture and salt contents were determined by standard methods [32, 51]. Water activities (*a*_w_) of grated cheese samples were measured using a Aqualab CX-2 (Decagon Devices, USA). Measurements of pH were performed by placing a surface electrode (Inlab 426, Mettler-Toledo, Glostrup, Denmark) connected to a pH meter (1120, Mettler-Toledo) directly on the cheese samples. Calibration of the electrode was performed in buffers with pH 4.01 and 7.00 (Radiometer, Brønshøj, Denmark).

### Repetitive Sequenced‐Based PCR (rep‐PCR)

Yeast and bacterial isolates were dereplicated using (GTG)5-PCR finger printing. Initially total DNA was extracted using InstaGene Matrix DNA extraction kit (Bio-Rad, Hercules, CA, USA) following the instructions of the manufacturer. Rep-PCR reaction was carried out in a 25-μl volume containing 1 U DreamTaq^TM^ DNA polymerase (Fermentas, St. Leon-Rot, Germany), 2.5 μl 10 × DreamTaq^TM^ Green Buffer containing 20 mM MgCl_2_ (Fermentas), 200 μM of each deoxynucleotide triphosphate (Fermentas), 0.8 μM of primer GTG_5_ (5′-GTG GTG GTG GTG GTG-3′) (DNA Technologies, Aarhus, Denmark), 1.5 μl of DNA template and sterile MilliQ water for adjustment of the volume to 25 μl. The PCR reaction was performed on a RoboCycler®Gradient 96 (Agilent Technologies, Santa Clara, CA, USA) using the following program: 5 min of initial denaturation at 94 °C, 30 cycles of 94 °C for 30 s, 45 °C for 60 s, 65 °C for 8 min followed by a final elongation step of 65 °C for 16 min and holding at 4 °C. The PCR products were separated by 1.5 % agarose gel electrophoresis in 1× TBE (90 mM Trizma base (Sigma), 90 mM Boric acid (Sigma), 2 mM EDTA (Merck, Darmstadt, Germany) pH8.0) (5 h, 140 V) using a Generuler 1 kb DNA ladder as reference (Fermentas). Following electrophoresis, gels were stained with ethidium bromide and photographed with UV transillumination (302 nm) using a Kodak EDAS 290 system (Eastman Kodak). Patterns were grouped based on the fraction of shared bands determined by Dice coefficient and clustering was calculated by the unweighted pair group algorithm with arithmetic averages (UPGMA).

### Sequencing of 16S and 26S rRNA Genes

Bacterial isolates were identified by sequencing of the 16S rRNA gene using following primers: 7f (5′-AGAGTTTGAT(C/T)(A/C)TGGCTCAG-3′) and 1510r (5′-ACGG(C/T)TACCTTGTTACGACTT-3′). Yeast isolates were identified by sequencing of the 26S rRNA gene using the following primers: NL-1 (5′-GCATATCAATAAGCGGAGGAAAAG-3′) and NL-4 (5′-GGTCCGTGTTTCAAGACGG-3′). Reactions were performed in an automatic thermal cycler (GeneAmp®PCR System 9700, Perkin-Elmer) under the following conditions: Initial denaturation at 95 °C for 5 min; 35 cycles of 95 °C for 1 min, 52 °C for 45 s and 72 °C for 1 min; final extension at 72 °C for 7 min and holding at 4 °C. PCR products were sent to a commercial sequencing facility (Macrogene Korea). The primers 7f and 1510r or NL-1 and NL4 were used in the sequencing reactions, respectively. Sequences were manually corrected and assembled by use of the software CLC Main Workbench 6.0 (Aarhus, Denmark). Bacterial and yeast sequences were compared to the sequences reported in EzTaxon and GenBank, respectively, using the BLAST (Basic Local alignment Search Tool) algorithm. From each rep-PCR group, at least the square root of the number of isolates was sequenced. The nucleotide sequences determined in this study have been assigned Genbank Accession Nos. JQ680412–JQ680469.

### DNA Extraction from Cheese Samples

Casein particles were removed from 40 ml of the 1:10 dilution by centrifugation (300×*g* for 10 min). The supernatant were transferred to a new tube, and cells were pelleted by centrifugation (5,000×*g* for 15 min) and washed once with 0.9 % (w/v) NaCl. DNA was extracted using GenElute™ Bacterial Genomic DNA Kit (NA2110; Sigma-Aldrich, St. Louis, MO, USA) following the instructions of the manufacturer.

### Denaturing Gradient Gel Electrophoresis

The V3 region of the 16S rRNA gene was amplified using the universal bacterial primers PRBA338fGC/PRUN518r [45]. Furthermore, an approximately 250-bp-long fragment of D1/D2 region of the 26S rRNA gene was amplified using the eukaryotic universal primers NL1GC/LS2 [9, 29]. The reaction mixture was as described by Nielsen et al. [44], and the thermocycling conditions as described in previous reports [45, 55]. The DGGE analysis was performed using the INGENY phorU (Ingeny International BV, the Netherlands). Polyacrylamide gels (8 % (wt/vol) acrylamide–bisacrylamide (37.5:1); Bio-Rad) in 1× TAE buffer (40 mM trizma base (Sigma), 20 mM acetic acid (Merck), 1 mM EDTA (Merck) pH 8.0) were prepared with a Bio-Rad Gradient Delivery System (Model 475, Bio-Rad) using solutions containing from 35 to 70 % denaturant [100 % denaturant corresponds to 7 M urea (ICN Biomedicals, Aurora, USA) and 40 % (vol/vol) formamide (Merck)]. Gels were run at 60 °C for 16 h at a constant voltage of 120 V. After electrophoresis, gels were stained with SYBR-GOLD (Molecular Probes, Eugene, OR, USA) for 2 h with mild shaking and photographed with UV transillumination (302 nm) using a Kodak EDAS 290 system (Eastman Kodak). The identity of selected DGGE bands was revealed by sequencing. DNA fragments from selected bands excised from the gels, re-amplified, the electrophoretic mobility relative to the fragment from which they were excised, was checked. In case of several bands on the DGGE gel, the target bands were excised from the gel again and analyzed by DGGE until a single band was obtained. The fragments were sequenced by Macrogene Korea. The sequences were assembled by use of CLC Main Workbench 6.0 (CLC bio, Aarhus, Denmark) and compared to the sequences in the GenBank using BLAST (http://blast.ncbi.nlm.nih.gov, January/2011).

### Statistical Analysis

To test whether there was a significant difference (95 % confidence level) between the cheese samples, a one-way ANOVA using Tukey HSD test was performed with JMP 8 (SAS Institute, Cary, NC, USA).

## Results

### Chemical Composition of the Cheeses

Table 1 shows the moisture content, NaCl content, NaCl-in-moisture contents, water activity (*a*_w_) and pH for three farmhouse cheeses and one industrial produced cheese. The moisture in the cheese core varied from 32 to 45 g/100 g cheese, whereas moisture on the cheese surfaces varied from 20 to 31 g/100 g cheese. The NaCl content ranged between 1.08 and 1.96 g/100 g cheese for interiors and between 0.93 and 1.40 g/100 g cheese for surfaces. For the individual cheeses, the NaCl-in-moisture content was significantly lower for interiors (3.03–4.65 % (w/v)) compared to surfaces (4.55–5.94 % (w/v)). The water activity (*a*_w_) of the interiors and surfaces varied between 0.824 and 0.876 but there were no significant difference. For all cheeses, core pH was significantly lower compared to surface pH. The core pH varied between 5.44 and 6.06, whereas surface pH varied between 6.44 and 7.28.

**Table 1:** Relevant characteristics of the surface and interior of Danish surface-ripened cheeses

Cheese	Moisture content^a^	NaCl content^a^	NaCl-in-moisture content^a^	Water activity^a^	pH^a^
	(g/100 g cheese)	(g/100 g cheese)	(%)	(*a*_w_)	
Dairy A					
Core	42 ± 0.23^B^	1.96 ± 0.0021^A^	4.65 ± 0.20^B^	0.824 ± 0.0014^D^	6.06 ± 0.070^D^
Surface	22 ± 0.92^E^	1.31 ± 0.0071^CD^	5.94 ± 0.23^A^	0.826 ± 0.0^D^	6.44 ± 0.095^C^
Dairy B					
Core	32 ± 0.49^D^	1.08 ± 0.0^E^	3.40 ± 0.053^CD^	0.838 ± 0.0021^CD^	5.74 ± 0.098^E^
Surface	21 ± 0.23^E^	1.24 ± 0.051^D^	5.82 ± 0.30^A^	0.852 ± 0.0021^BC^	6.56 ± 0.076^BC^
Dairy C					
Core	37 ± 0.60^C^	1.40 ± 0.0099^B^	3.84 ± 0.036^C^	0.840 ± 0.0091^CD^	5.71 ± 0.098^E^
Surface	20 ± 0.24^E^	0.93 ± 0.0032^F^	4.62 ± 0.040^B^	0.842 ± 0.0057^CD^	6.69 ± 0.14^B^
Dairy D					
Core	45 ± 0.035^A^	1.36 ± 0.015^BC^	3.03 ± 0.031^D^	0.872 ± 0.0042^AB^	5.44 ± 0.047^F^
Surface	31 ± 1.1^D^	1.40 ± 0.0021^B^	4.55 ± 0.16^B^	0.876 ± 0.011^A^	7.28 ± 0.089^A^

Dairy A: farmhouse producing cheese of the Havarti type from raw milk, ripened for 12 weeks. Dairy B: farmhouse producing cheese of the Danbo type from pasteurised milk, ripened for 6 weeks. Dairy C: farmhouse producing cheese of the Havarti type from pasteurised milk, ripened for 12 weeks. Dairy D: industrial dairy producing cheese of the Danbo type from pasteurised milk, ripened for 6 weeks

^a^Values in same column not marked by same superscript capitals are significantly different using one-way ANOVA with Tukey HSD test (≥95 % confidence)

### Microbial Cell Counts

The bacterial and yeast counts from the cheese interior and surface are shown in Table 2. The interior bacterial counts for cheese A (3.6×10^7^ CFU g^−1^ on GM17, 5.6×10^7^ CFU g^−1^ on MRS pH 6.2 and 2.8×10^6^ CFU g^−1^ on MRS pH 5.4) were significantly higher than the counts for cheese B, C and D varying between 3.1 × 10^5^ and 5.7 × 10^5^ CFU g^−1^ on GM17, between 3.5 × 10^5^ and 6.6 × 10^5^ CFU g^−1^ on MRS pH 6.2 and between 6.1 × 10^3^ and 7.4 × 10^5^ CFU g^−1^ on MRS pH 5.2. Concerning the surface bacterial counts, generally no significant differences between the cheeses were observed as they varied between 1.4 × 10^8^ and 8.1 × 10^8^ CFU cm^−2^ on TSA without added NaCl and between 2.0×10^8^ and 6.0×10^8^ CFU cm^−2^ on TSA supplemented with 4.0 % (w/v) NaCl. The yeast surface counts were consistently lower than the bacterial counts. The yeast count for cheese C (3.7 × 10^6^ CFU cm^−2^) was significantly higher than the other cheeses varying between 1.2×10^5^ and 7.4 ×10^5^ CFU cm^−2^. As expected, no yeasts were identified on MYGP from the interior of the cheeses (results not shown).

**Table 2:** Bacterial and yeast counts for Danish surface-ripened cheeses

	Dairy A^a^	Dairy B^a^	Dairy C^a^	Dairy D^a^
Interior (CFU g^−1^)				
Lactic acid bacteria				
GM17	3.6×10^7^ ± 1.4×10^7,A^	3.1×10^5^ ± 2.8×10^4,B^	4.9×10^5^ ± 5.0×10^4,B^	5.7x 10^5^ ± 9.9×10^4,B^
MRS pH 6.2	5.6×10^7^ ± 1.5×10^7,A^	4.0×10^5^ ± 2.7×10^5,B^	3.5×10^5^ ± 1.4×10^5,B^	6.6×10^5^ ± 2.1×10^4,B^
MRS pH5.4	2.8×10^6^ ± 3.5×10^5,A^	6.1×10^3^ ± 7.1×10^2,B^	4.5×10^3^ ± 1.6×10^3,B^	7.4×10^5^ ± 1.6×10^5,B^
Surface (CFU cm^−2^)				
Aerobic bacteria				
TSA 0 % (w/v) NaCl	8.1×10^8^ ± 3.0×10^8,A^	1.4×10^8^ ± 2.8×10^8,A^	5.3×10^8^ ± 2.6×10^8,A^	6.3×10^8^ ± 1.8×10^8,A^
TSA 4 % (w/v) NaCl	5.1×10^8^ ± 5.1×10^7,A^	2.0×10^8^ ± 2.4×10^7,B^	6.0×10^8^ ± 3.5x 10^7,A^	5.2×10^8^ ± 2.9×10^8,A^
Yeasts				
MYGP	7.4×10^5^ ± 1.7×10^5,B^	1.2×10^5^ ± 2.4×10^4,C^	3.7×10^6^ ± 1.4×10^5,A^	4.8×10^5^ ± 1.3×10^5BC^

Dairy A: farmhouse producing cheese of the Havarti type from raw milk, ripened for 12 weeks. Dairy B: farmhouse producing cheese of the Danbo type from pasteurised milk, ripened for 6 weeks. Dairy C: farmhouse producing cheese of the Havarti type from pasteurised milk, ripened for 12 weeks. Dairy D: industrial-scale dairy producing cheese of the Danbo type from pasteurised milk, ripened for 6 weeks.

^a^Values in same row not marked by same superscript capitals are significantly different using one-way ANOVA with Tukey HSD test (≥95 % confidence)

### Grouping and Identification of the Cheese Microbiota

**Figure 1:**
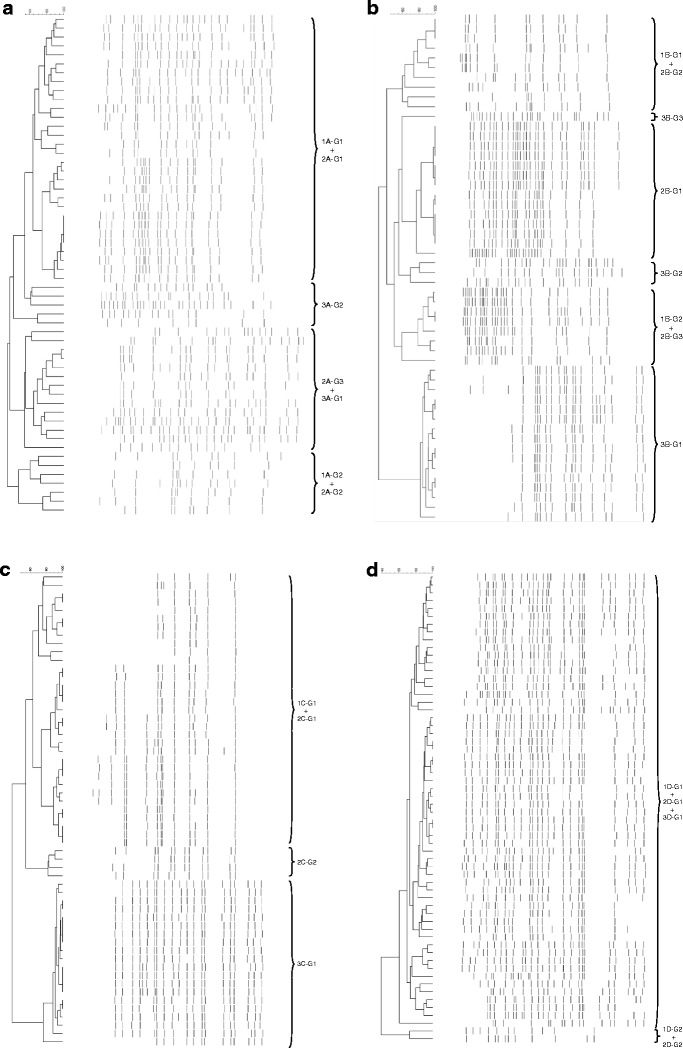
Dendrogram obtained from GTG_5_-PCR fingerprint of the interior bacterial isolates from GM17 and MRS with pH 6.2 and 5.4. **a** Dairy A, **b** Dairy B, **c** Dairy C and **d** Dairy D. The identification of the groups can be seen in Table 3

**Figure 2:**
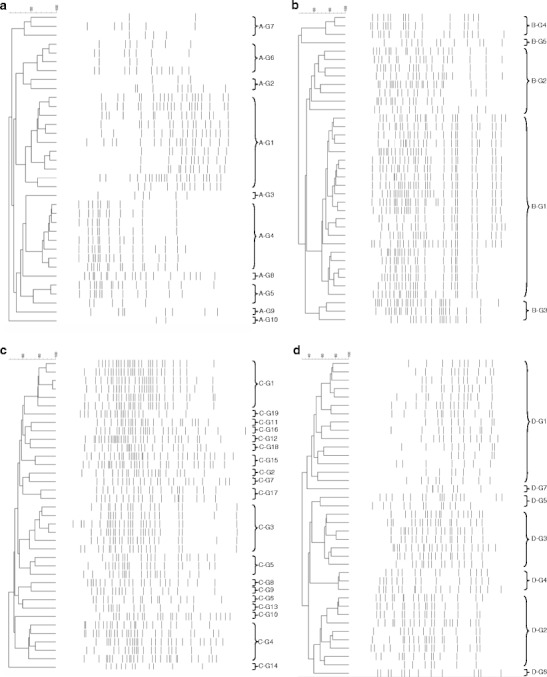
Dendrogram obtained from GTG_5_-PCR fingerprint of the surface bacterial isolates from TSA added 4.0 % (w/v) NaCl. **a** Dairy A, **b** Dairy B, **c** Dairy C and **d** Dairy D. The identification of the groups can be seen in Table 4

**Figure 3:**
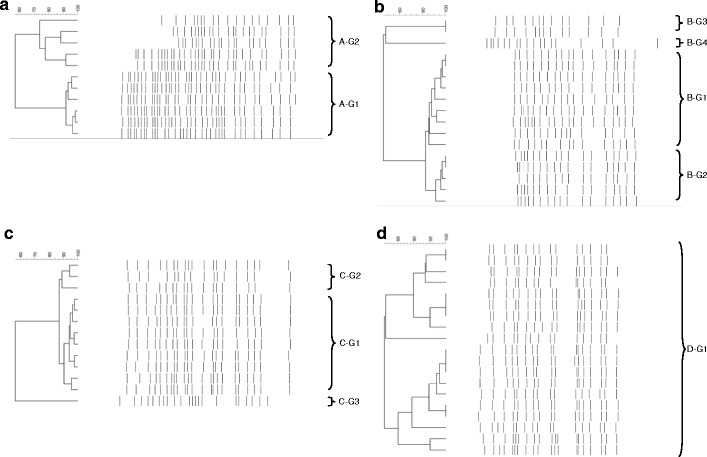
Dendrogram obtained from GTG_5_-PCR fingerprint of the surface yeast isolates from MYGP. **a** Dairy A, **b** Dairy B, **c** Dairy C and **d** Dairy D. The identification of the groups can be seen in Table 5

Detection of Rep-PCR profiles was proved to be a reliable and rapid method for grouping of both bacterial and yeast isolates (Figs. 1, 2 and 3). For the majority of the groups, variations in rep-PCR profiles were observed indicating that strain variation occurs within the groups. Similarly, different groups containing identical species is due to strain variations. Representative isolates from each rep-PCR group were identified by sequencing of rRNA genes. Most sequenced bacterial and yeast isolates showed high similarities (>99 %) to sequences in EzTaxon and Genbank, respectively (Tables 3, 4 and 5).

**Table 3:** Identification of the interior bacterial community by culture dependent isolation followed by16S rRNA gene sequencing

Rep-PCR group	Sequence length (bp)	Similarity (%)	Closest phylogenetic affiliation in EzTaxon	Distribution (%)	GenBank accession number
*GM17 (30 °C)*					
Dairy A					
1A-G1	1,364	100	*Leuconostoc mesenteroides*	74	JQ680412
1A-G2	1,375	99.9	*Lactococcus lactis* subsp. *lactis*	26	JQ680413
Dairy B					
1B-G1	1,410	100	*Lactococcus lactis* subsp. *lactis*	53	JQ680414
1B-G2	1,399	99.6	*Leuconostoc pseudomesenteroides*	47	JQ680415
Dairy C					
1C-G1	1,340	100	*Lactococcus lactis* subsp*. lactis*	100	JQ680416
Dairy D					
1D-G1	1,441	100	*Lactobacillus paracasei*	95	JQ680417
1D-G2	1,415	99.9	*Lactococcus lactis* subsp*. cremoris*	5	JQ680418
*MRS (pH 6.2, 30 °C)*					
Dairy A					
2A-G1	1,421	100	*Leuconostoc mesenteroides*	80	JQ680419
2A-G2	1,385	100	*Lactococcus lactis* subsp. *lactis*	10	JQ680420
2A-G3	1,382	100	*Lactobacillus paracasei*	10	JQ680421
Dairy B					
2B-G1	1,425	99.4	*Lactobacillus oligofermentans*	82	JQ680422
2B-G2	1,320	100	*Lactococcus lactis* subsp. *lactis*	12	JQ680423
2B-G3	1,361	99.4	*Leuconostoc pseudomesenteroides*	6	JQ680424
Dairy C					
2C-G1, 2C-G2	1,409	100	*Lactococcus lactis* subsp. *lactis*	100	JQ680425
Dairy D					
2D-G1	1,435	100	*Lactobacillus paracasei*	95	JQ680426
2D-G2	1,407	100	*Lactococcus lactis* subsp*. cremoris*	5	JQ680427
*MRS (pH 5.4, 37 °C)*					
Dairy A					
3A-G1	1,405	99.9	*Lactobacillus paracasei*	71	JQ680428
3A-G2	1,448	100	*Lactobacillus parabuchneri*	29	JQ680429
Dairy B					
3B-G1	1,432	99.9	*Lactobacillus brevis*	80	JQ680430
3B-G2	1,428	99.0	*Lactobacillus farciminis*	15	JQ680431
3B-G3	1,409	100	*Enterococcus dispar*	5	JQ680432
Dairy C					
3C-G1	1,411	100	*Lactobacillus paracasei*	100	JQ680433
Dairy D					
3D-G1	1,438	100	*Lactobacillus paracasei*	100	JQ680434

**Table 4:** Identification of the surface bacterial community by culture dependent isolation followed by 16S rRNA gene sequencing

Rep-PCR group	Sequence length (bp)	Similarity (%)	Closest phylogenetic affiliation in EzTaxon	Distribution	GenBank accession number
Dairy A					
A-G1, A-G2, A-G3	1,373	99.6	*Brachybacterium alimentarum*	40	JQ680435
A-G4, A-G5	1,348	99.1	*Corynebacterium casei*	31	JQ680436
A-G6, AG-7, AG-8	1,374	98.8	*Brevibacterium permense*	23	JQ680437
A-G9	1,371	99.7	*Leucobacter albus*	3	JQ680438
A-G10	1,380	99.2	*Microbacterium gubbeenense*	3	JQ680439
Dairy B					
B-G1	1,371	100	*Corynebacterium variabile*	59	JQ680440
B-G2	1,395	99.4	*Brevibacterium linens*	22	JQ680441
B-G3	1,387	99.3	*Corynebacterium casei*	8	JQ680442
B-G4	1,420	100	*Staphylococcus saprophyticus*	8	JQ680443
B-G5	1,390	99.9	*Halomonas alkaliphila*	3	JQ680444
Dairy C					
C-G1, C-G2	1,369	99.3	*Corynebacterium casei*	19	JQ680445
C-G3	1,398	100	*Staphylococcus equorum*	16	JQ680446
C-G4	1,397	99.6	*Brevibacterium aurantiacum*	14	JQ680447
C-G5, C-G6	1,390	100	*Microbacterium gubbeenense*	11	JQ680448
C-G7, C-G8, C-G9, C-G10	1,388	100	*Agrococcus casei*	11	JQ680449
C-G11, C-G12, C-C13, C-G14	1,317	98.4	*Brachybacterium* spp.	11	JQ680450
C-G15, C-G16	1,381	100	*Corynebacterium variabile*	8	JQ680451
C-G17	1,397	99.9	*Alcaligenes faecalis*	5	JQ680452
C-G18	1,303	99.8	*Proteus vulgaris*	3	JQ680453
C-G19	1,427	99.8	*Enterococcus* spp.	3	JQ680454
Dairy D					
D-G1	1,381	100	*Corynebacterium variabile*	39	JQ680455
D-G2	1,423	100	*Staphylococcus equorum*	24	JQ680456
D-G3	1,359	99.3	*Corynebacterium casei*	18	JQ680457
D-G4	1,419	100	*Staphylococcus saprophyticus*	8	JQ680458
D-G5	1,422	100	*Marinilactibacillus psychrotolerans*	5	JQ680459
D-G6	1,429	100	*Bavariicoccus seileri*	3	JQ680460
D-G7	1,392	99.7	*Micrococcus* spp.	3	JQ680461

Bacterial isolates have been isolated from TSA with 4.0 % (w/v) NaCl incubated at 30 °C

**Table 5:** Identification of the surface yeast community by culture dependent isolation followed by 26S rRNA gene sequencing

Rep-PCR group	Sequence length (bp)	Similarity (%)	Closest phylogenetic affiliation in GenBank	Distribution (%)	GenBank accession number
Dairy A					
A-G1	538	100	*Yarrowia lipolytica*	55	JQ680462
A-G2	568	98.8	*Scopulariopsis brevicaulis* ^a^	45	JQ680463
Dairy B					
B-G1, BG2	556	100	*Geotrichum* spp	82	JQ680464
B-G3	538	100	*Kluyveromyces marxianus*	12	JQ680465
B-G4	576	100	*Debaryomyces hansenii*	6	JQ680466
Dairy C					
C-G1, C-G2	581	100	*Debaryomyces hansenii*	92	JQ680467
C-G3	557	98.7	*Geotrichum* spp	8	JQ680468
Dairy D					
D-G1	597	100	*Debaryomyces hansenii*	100	JQ680469

Yeast isolates have been isolated from MYGP incubated at 25 °C

^a^ *Scopulariopsis brevicaulis* is classified as a mould

Figure 1 shows the grouping of the interior bacterial isolates from GM17 and MRS with pH 6.2 and 5.4. The interior bacterial isolates from the cheeses from dairies A and B were divided into more groups (four and six groups, respectively) than the interior bacterial isolates from the cheeses from dairies C and D (three and two groups, respectively). The cheeses from dairies A and C were primarily dominated by the LAB starter cultures *Leuconostoc mesenteroides* and *Lactococcus lactis* subsp. *lactis* (Table 3). Furthermore, the cheeses from dairies A and C were dominated by the non-starter lactic acid bacteria (NSLAB) *Lactobacillus paracasei*, and for the cheese from dairy A, a minor group consisting of the NSLAB *Lb. parabuchneri* was found. The interior bacterial isolates from the cheeses from dairies B and D were primarily dominated by NSLAB. The cheese from dairy B was dominated by a range of NSLAB including *Lb. brevis*, *Lb. oligofermentans* and *Lb. farminis*, whereas the cheese from dairy D was exclusively dominated by the NSLAB *Lb. paracasei*. Additionally, minor groups of the LAB starter cultures *Lc. lactis* subsp. *lactis* and *Lc. lactis* subsp. *cremoris* were found on the cheeses from dairies B and D, respectively.

Figure 2 shows the grouping of the surface bacterial isolates from TSA supplemented with 4.0 % (w/v) NaCl. Similar results were obtained for the surface bacterial isolates from TSA without NaCl (results not shown). Generally, the surface bacterial isolates were divided into more groups compared to the interior bacterial isolates, indicating that the surface microbiota is more complex than the interior bacterial microbiota. The surface bacterial isolates from cheeses from dairy A and C were divided into a higher number of groups (ten and 19 groups, respectively) than the surface bacterial isolates from cheeses from dairy B and D (five and seven groups, respectively). However, identifications showed the ten groups found on the cheese from dairy A only represented five different species, whereas the 19 groups found on the cheese from dairy C represented ten different species indicating strain variation as mentioned above (Table 4). For dairy B five different species were identified whereas for dairy D seven different species were identified.

Most species were Gram-positive Actinobacteria with *Corynebacterium casei* and/or *C. variabile* as the predominant (Table 4). Additionally, the cheese from dairy A was dominated by high of numbers of *Brachybacterium alimentarum*. Various *Brevibacterium* species were found on the cheeses from the farmhouses (dairies A, B and C). *B. permense* was found on the cheese from dairy A, *B. linens* was found on the cheese from dairy B and *B. aurantiacum* was found on the cheese from dairy C. *Brevibacterium* spp. could not be isolated on the cheese from dairy D. Furthermore, a number of coagulase negative staphylococci were found, i.e., *Staphylococcus saprophyticus* on the cheeses from dairies B and D, and *Staph. equorum* on the cheeses from dairies C and D. Finally, a number of Gram-negative bacteria species including *Proteus vulgaris* and *Alcaligenes faecalis* was found on the cheese from dairy C.

Figure 3 shows the grouping of the surface yeast microbiota. The yeast surface microbiota on the three farmhouse cheeses consisted of two to four groups, whereas the cheese produced at the industrial dairy (dairy D) consisted of only one single group. The cheese from dairy A was equally dominated by *Yarrowia lipolytica* and *Scopulariopsis brevicaulis*. The yeast microbiota on cheese from dairy B was primarily dominated by *Geotrichum* spp., however, *Kluyveromyces marxianus* and *Debaryomyces hansenii* were additionally found in minor amounts. The cheese from dairy C was dominated by *D*. *hansenii* followed by a minor group of *Geothrichum* spp. Finally, the cheese from dairy D was entirely dominated by *D*. *hansenii*.

### Denaturing Gradient Gel Electrophoresis

**Figure 4:**
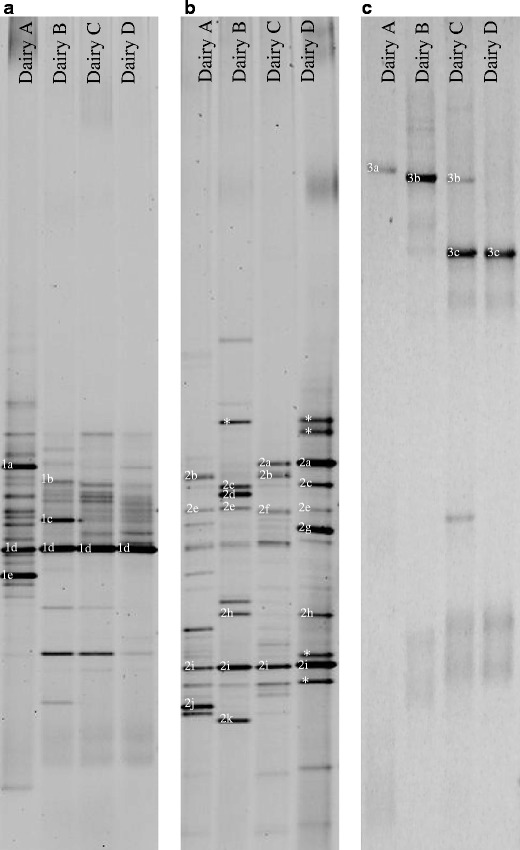
DGGE profiles for cheeses from dairies A, B, C and D. **a** Bacteria from interior of the cheeses, **b** bacteria from the surface of the cheeses and **c** yeasts from the surface of the cheeses. Bands were identified as 1a: *Leuconostoc mesenteroides,* 1b: *Lactobacillus brevis,* 1c: *Lactobacillus oligofermentans,* 1d: *Lactococcus lactis* subsp*. lactis,* 1e: *Streptococcus thermophilus,* 2a: *Staphylococcus equorum,* 2b: *Vagococcus carniphilus,* 2c: *Bavariicoccus seileri,* 2d: *Lactobacillus curvatus,* 2e: *Psychrobacter* spp.*,* 2f: *Proteus vulgaris,* 2 g: *Marinilactibacillus psychrotolerans,* 2 h: *Corynebacterium variabile,* 2i: *Corynebacterium casei,* 2j: *Brachybacterium* sp., 2 k: *Brevibacterium linens,* 3a: *Yarrowia lipolytica,* 3b: *Geotrichum* spp., 3c: *Debaryomyces hansenii*. *Strong bands that were not identified

Culture dependent isolation followed by genotypic identifications was basically confirmed by the culture-independent method, DGGE (Fig. 4). Additionally, in the sample from the interior of the cheese from dairy A, DGGE band with strong intensity was identified as *Streptococcus thermophilus*. Furthermore, DGGE bands with strong intensities from the cheese surface samples were found to represent *Vagococcus carniphilus* (the cheeses from dairies A, B and D), *Psychrobacter* spp. (the cheeses from dairies A and C) and *Lb. curvatus* (the cheese from dairy B) indicating that these bacterial species may play a role in cheese ripening, even though they were not found by the culture dependent approach. Unfortunately, several major DGGE bands in the samples from the cheese surface could not be successfully identified.

## Discussion

In addition to a culture dependent approach, a culture independent approach using DGGE was included for identification of the microbial microbiota in Danish cheeses. Most significantly, the DGGE analysis found that *Streptococcus thermophilus* is present in raw milk cheese from dairy A, even though this thermophilic lactic acid bacterium was not included in the mesophilic LAB starter culture used for production of the raw milk cheese from dairy A. No culturable *Str*. *thermophilus* isolates was isolated from GM17 agar incubated at 37 °C (data not shown). In the study by Masoud et al. [38], *Str*. *thermophilus* was similarly detected by DGGE analysis without being added as part of the LAB starter culture. However, Masoud et al. [38] excluded that *Str*. *thermophilus* originates from the raw milk, as it was not identified in the DGGE profile of the raw milk. Even though the source of *Str*. *thermophilus* remains unknown, it is likely that *Str*. *thermophilus* may play an important role in milk acidification and cheese ripening as previously reported [36, 47, 48]. Concerning the surface microbiota, which was found to be the most complex microbiota with the highest number of species, some limitations by the DGGE method was observed as several DGGE bands with strong intensity could not be successfully sequenced, which suggests that PCR products of several bacterial species comigrated as previously reported by Sekiguchi et al. [53].

Flavour formation may be enhanced during ripening, if strains that lyse rapidly are selected [10, 18]. A difference in autolysis can be seen between the two subspecies of *Lactococcus lactis*. *Lactococcus lactis* subsp. *lactis* survives better in cheese than *Lc*. *lactis* subsp. *cremoris* [8]. This fact even though not examined in the present study suggests that the level of autolysis of the latter is the highest and thus explains why *Lc*. *lactis* subsp. *cremoris* was generally not found in the cheeses at the end of ripening, even though it was added as a part of the primary LAB starter culture. Contrary, the NSLAB *Lactobacillus paracasei* was found in three out of four cheeses. This confirms the findings by Antonsson et al. [1], who found *Lb*. *paracasei* to be the main NSLAB in several Danbo cheeses.

During the last decades, there has been an increasing interest in using surface-ripening cultures for cheese production to ensure the presence of desirable microorganisms necessary for the ripening process. However, recent investigations have revealed that these commercial ripening cultures do not establish well on the cheese surfaces [20, 46, 50]. These observations were confirmed in the present study as *Brevibacterium linens* was found to be subdominant compared to other bacteria on the farmhouse cheeses, and no *Brevibacterium* spp. was found on the cheese from the industrial dairy. Also, the variations seen at the strain level by the rep-PCR technique indicate that several indigenous cultures take part in the maturation process. This finding underlines that microorganisms selected as starter cultures expressing defined functions often behave differently in complex microbial communities or often cannot compete with the indigenous microbiota. Several studies indicate that the microbial succession during cheese ripening depends on adaption of the cultures to stress conditions such as high NaCl concentrations, low water activity [22, 40, 46]. Most recently, growth of aerobic ripening bacteria at cheese surfaces has been shown to be limited by the availability of iron [39].

*B. linens* was usually reported to be the most important bacterial species associated with cheese surfaces [13, 28, 49]. In the present study, various *Brevibacterium* spp. were found on the cheeses from the farmhouses (dairies A, B and C). *B. linens* and *B. aurantiacum*, found on the cheeses from dairies B and C, respectively, have been used for a long time as ripening cultures by the dairy industry [16], whereas the soil bacteria *B. permense* found on the cheese from dairy A, to our knowledge, has not previously been found on cheese.

Several studies have now shown that *Corynebacterium* spp. is the most dominant bacterial species on surface-ripened cheeses [3, 6, 34]. *C. casei* and/or *C. variabile* were found in the present study to be the predominant bacterial species on the surfaces of the four cheeses and thus potentially important during cheese ripening. These data confirm that strains of *Corynebacterium* spp. are candidates as ripening cultures for production of surface-ripened cheeses.

Since the study by Maoz et al. [34], several authors have reported a Gram-negative microbiota on surface-ripened cheeses. First, the Gram-negative bacterial microbiota was associated with hygienic problems, but more recently an interest in their role in flavour production has been reported [11]. In the present study, a significant Gram-negative bacterial microbiota consisting of *Proteus vulgaris* and *Alcaligenes faecalis* was found on cheese from dairy C. A previous study has focused on *P*. *vulgaris* as cheese ripening culture [11]. *P*. *vulgaris* was found to produce important flavour notes including aldehydes and acids, but influenced other surface-ripening cultures negatively. *A*. *faecalis*, which is found in soil, water, and environments in association with humans and generally considered non-pathogenic [19], has also previously been found on Livarot cheese [30].

The present study confirms the presence of the marine bacteria *Marinilactibacillus psychrotolerans* on cheese as this species was found on the surface of cheese D. Both French and German cheeses have previously been reported to contain *M. psychrotolerans* [14, 34]. It was suggested that *M. psychrotolerans* is transferred to the cheeses from the marine environments via sea salt. However, its potential role in cheese ripening remains unknown.

Yeasts play an essential role in deacidification of the cheese surface due to lactate assimilation and production of alkaline metabolites, e.g., ammonia [23, 46], which is prerequisite for development of the less acid tolerant bacterial microbiota [4]. The present study confirms that *Debaryomyces hansenii* and *Geotrichum* spp. are the dominating yeast species on surface-ripened cheeses. *D*. *hansenii* was found to be the dominating yeast species on the cheeses from dairies C and D, whereas *Geotrichum* spp. was found to dominate on the cheese from dairy B. On the cheese from dairy A, *Yarrowia lipolytica* was the dominating yeast species. *Y*. *lipolytica* is a naturally developing yeast species on cheese surfaces, and has in some cases been shown to rapidly outnumber other yeast species including *D*. *hansenii* and *Geotrichum* spp. [33]. This may explain why the latter yeast species are not found on the cheese from dairy A. Finally, the filamentous fungus *Scopulariopsis brevicaulis* was found in a high number on cheese A. *S. brevicaulis* has previously been found in Danish cheese [52], and has been subject to spoilage of cheeses due to its high proteolytic activity resulting in ammonia production and its production of arsenical compounds, e.g., diethylarsine, which has a very characteristic garlic-like odour [5].

In conclusion, the interior bacterial microbiota of the Danish cheeses consisted of LAB starter cultures as well as one or more NSLAB. Noticeable for the bacterial community of the cheese surfaces was that *B*. *linens* did not establish well on the cheeses, even though it was used as ripening culture. Contrarily *C. casei* and/or *C. variabile* were predominant, whereas the surface yeast microbiota resembled that otherwise stated in literature. Generally, the Danish cheeses produced at farmhouses had a more complex microbiota comprising of a higher number of bacterial strains both in the interior and on the surface compared to the Danish cheese produced at the industrial dairy. Culture dependent identifications were basically confirmed by the culture independent method DGGE, even though the latter technique proved the presence of additional cultures including *Str. thermophilus* in cheese interiors as well as *Vagococcus carniphilus*, *Psychrobacter* spp. and *Lb. curvatus* on cheese surfaces. Due to the limited number of cheeses included, further studies have to be performed to confirm the composition of the microbial ecology in Danish cheeses. Knowledge on the microbial community of cheeses may be used for improving process and ripening conditions in order to enhance the quality and consistency of the final product. Finally, the cultures isolated can potentially be used as starter or ripening cultures for production of Danbo- and Havarti-type cheeses.
